# Causal relationship between obesity and iron deficiency anemia: a two-sample Mendelian randomization study

**DOI:** 10.3389/fpubh.2023.1188246

**Published:** 2023-06-16

**Authors:** Tingting Wang, Qi Gao, Yuanyuan Yao, Ge Luo, Tao Lv, Guangxin Xu, Mingxia Liu, Jingpin Xu, Xuejie Li, Dawei Sun, Zhenzhen Cheng, Ying Wang, Chaomin Wu, Ruiyu Wang, Jingcheng Zou, Min Yan

**Affiliations:** ^1^Department of Anesthesiology, Second Affiliated Hospital, Zhejiang University School of Medicine, Hangzhou, China; ^2^Department of Anesthesiology, The Fourth Affiliated Hospital, International Institutes of Medicine, Zhejiang University School of Medicine, Yiwu, China

**Keywords:** obesity, iron deficiency anemia, Mendelian randomization, causal relationship, two-sample

## Abstract

**Background:**

Observational studies have suggested an association between obesity and iron deficiency anemia, but such studies are susceptible to reverse causation and residual confounding. Here we used Mendelian randomization to assess whether the association might be causal.

**Methods:**

Data on single-nucleotide polymorphisms that might be associated with various anthropometric indicators of obesity were extracted as instrumental variables from genome-wide association studies in the UK Biobank. Data on genetic variants in iron deficiency anemia were extracted from a genome-wide association study dataset within the Biobank. Heterogeneity in the data was assessed using inverse variance-weighted regression, Mendelian randomization Egger regression, and Cochran's Q statistic. Potential causality was assessed using inverse variance-weighted, Mendelian randomization Egger, weighted median, maximum likelihood and penalized weighted median methods. Outlier SNPs were identified using Mendelian randomization PRESSO analysis and “leave-one-out” analysis.

**Results:**

Inverse variance-weighted regression associated iron deficiency anemia with body mass index, waist circumference, trunk fat mass, body fat mass, trunk fat percentage, and body fat percentage (all odds ratios 1.003–1.004, *P* ≤ 0.001). Heterogeneity was minimal and no evidence of horizontal pleiotropy was found.

**Conclusion:**

Our Mendelian randomization analysis suggests that obesity can cause iron deficiency anemia.

## Introduction

Obesity is an energy metabolism disorder that results in excessive fat storage and that can lead to physical and psychological problems (1). The World Health Organization calls obesity a chronic disease and a major threat to public health, in part because it increases the risk of cardiovascular disease (2), diabetes mellitus, and cancer (3).

Obesity has been linked to another global public health problem, iron deficiency anemia (4, 5). In low- and middle-income countries, iron deficiency anemia is one of the five most frequent causes of chronic disability (6). Observational studies have provided strong evidence of an association between obesity and iron deficiency anemia in Israel (7), Turkey (8), Taiwan (9, 10), Bangladesh (11) and the US (12, 13). A study on the interaction between body mass index and iron deficiency anemia showed that overweight and obese women had a 10.11 (95%CI: 1.267–80.797) times greater risk of iron deficiency anemia (9). Another study conducted in 525 women of reproductive age showed that the prevalence with iron deficiency anemia was 43.0% (11). On the other hand, some observational studies have reported no significant association (14–16), while an observational study in Colombia suggested that overweight and obesity were associated with *lower* risk of iron deficiency anemia than normal-weight women (17). And another study also indicated that no correlation was found between body mass index and iron deficiency anemia, which showed that only 13.4% of obese women and 17.1% of normal-weight women had iron deficiency anemia (OR = 0.75; 95% CI:0.39–1.49, *P* > 0.05) (14). This discrepancy in the literature, combined with the vulnerability of observational studies to reverse causation and residual confounding (18), led us to seek a more rigorous approach to assessing whether obesity might cause iron deficiency anemia.

We turned to Mendelian randomization (MR) as a more robust method for inferring causality than conventional observational studies. The greater robustness is thought to arise from the fact that genotypes are defined at conception and are generally not associated with conventional confounders in observational studies (18, 19). MR involves testing for a causal relationship between exposure (in our case, obesity) and outcome (iron deficiency anemia) using genetic markers (single-nucleotide polymorphisms, SNPs) associated with the exposure (20). We therefore applied two-sample MR to data from large-scale genome-wide association studies that explored links between anthropometric traits of obesity and iron deficiency anemia.

## Methods

### Data sources and selection of instrumental variables

We extracted data on SNPs and the following seven obesity-linked anthropometric traits from the UK Biobank, a prospective cohort study involving more than half a million people in the UK aged 40–69 years (21): body mass index, waist circumference, hip circumference, trunk fat mass, whole-body fat mass, trunk fat percentage, and body fat percentage (22, 23) (Table 1). We included data from 361,194 individuals diagnosed with iron deficiency anemia in the present study.

**Table 1:** Characteristics of the eight genome-wide association studies used in the present work.

**Variable**	***n***	**no. SNPs**	Consortium^*^	**Source**
**Exposure variables: anthropometric traits**				
Body mass index	681,275	2,336,260	GIANT	PubMed ID: 30124842
Waist circumference	462,166	9,851,867	MRC-IEU	IEU OpenGWAS project (ukb-b-9405)
Hip circumference	462,117	9,851,867	MRC-IEU	IEU OpenGWAS project (ukb-b-15590)
Trunk fat mass	454,588	9,851,867	MRC-IEU	IEU OpenGWAS project (ukb-b-20044)
Whole-body fat mass	454,137	9,851,867	MRC-IEU	IEU OpenGWAS project (ukb-b-19393)
Trunk fat percentage	454,613	9,851,867	MRC-IEU	IEU OpenGWAS project (ukb-b-16407)
Body fat percentage	454,633	9,851,867	MRC-IEU	IEU OpenGWAS project (ukb-b-8909)
**Outcome variable**				
Iron deficiency anemia	3,222 cases, 357,972 controls	11,553,874	Not applicable	IEU OpenGWAS project (ukb-d-D50)

SNP, single-nucleotide polymorphism. ^*^The samples in all consortia were from Europe, so predominantly Caucasian.

Data on obesity-linked anthropometric traits and SNPs in individuals were obtained from genome-wide association studies within the UK Biobank as described in Supplementary Table 1. Publicly available summary data on body mass index and 2,336,260 SNPs were obtained from 681,275 individuals of European ancestry in the “Genetic investigation of anthropometric traits” consortium (24). Data were extracted, from genome-wide association studies in the MRC-IEU consortium (Table 2), for waist circumference from 462,166 individuals; hip circumference, 462,117 individuals; trunk fat mass, 454,588 individuals; whole-body fat mass, 454,137 individuals; trunk fat percentage, 454,613 individuals; and body fat percentage, 454,633 individuals.

**Table 2:** Mendelian randomization analysis of casual associations between obesity-related anthropometric traits and iron deficiency anemia.

**Trait**	**no. SNPs**	**Inverse variance-weighted analysis**				**MR-Egger analysis**					
		**OR (95%CI)**	***P***	**Q statistic**	***P***	**OR (95%CI)**	***P***	**Q statistic**	***P***	**Intercept**	***P***
Body mass index	492	1.003 (1.001–1.005)	< 0.001	564.867	0.012	1.002 (0.997–1.006)	0.447	564.382	0.011	2.249 × 10^−5^	0.517
Waist circumference	360	1.003 (1.002–1.005)	< 0.001	365.481	0.395	1.004 (0.998–1.009)	0.197	365.470	0.381	4.233 × 10^−6^	0.919
Hip circumference	405	1.001 (1.000–1.003)	0.088	490.861	0.002	1.000 (0.995–1.004)	0.874	490.004	0.002	3.481 × 10^−5^	0.402
Trunk fat mass	404	1.003 (1.002–1.005)	< 0.001	452.489	0.045	1.001 (0.997–1.006)	0.525	451.735	0.044	3.350 × 10^−5^	0.413
Whole-body fat mass	419	1.003 (1.001–1.005)	< 0.001	469.997	0.040	1.003 (0.998–1.007)	0.196	469.992	0.037	2.564 × 10^−6^	0.949
Trunk fat percentage	374	1.003 (1.001–1.005)	< 0.001	407.007	0.109	1.002 (0.996–1.008)	0.442	406.942	0.103	1.170 × 10^−5^	0.807
Body fat percentage	382	1.004 (1.002–1.006)	< 0.001	382.256	0.458	1.006 (1.000–1.013)	0.057	382.256	0.458	3.100 × 10^−5^	0.496
		**Weighted median analysis**		**Maximum likelihood analysis**		**Penalized weighted median analysis**	
		**OR (95%CI)**	***P***	**OR (95%CI)**	***P***	**OR (95%CI)**	***P***
Body mass index	492	1.003 (1.000–1.005)	0.029	1.003 (1.001–1.004)	< 0.001	1.003 (1.000–1.006)	0.023
Waist circumference	360	1.003 (1.000–1.006)	0.063	1.003 (1.002–1.005)	< 0.001	1.003 (1.000–1.006)	0.077
Hip circumference	405	1.002 (1.000–1.005)	0.081	1.001 (1.000–1.003)	0.060	1.002 (1.000–1.005)	0.076
Trunk fat mass	404	1.003 (1.001–1.006)	0.007	1.003 (1.002–1.005)	< 0.001	1.003 (1.001–1.006)	0.008
Whole-body fat mass	419	1.003 (1.001–1.006)	0.013	1.003 (1.002–1.005)	< 0.001	1.003 (1.005–1.006)	0.017
Trunk fat percentage	374	1.003 (1.001–1.006)	0.006	1.003 (1.001–1.005)	< 0.001	1.003 (1.002–1.007)	0.002
Body fat percentage	382	1.005 (1.002–1.008)	0.004	1.004 (1.002–1.006)	< 0.001	1.004 (1.001–1.008)	0.003

CI, confidence interval; MR, Mendelian randomization; OR, odds ratio; SNP, single-nucleotide polymorphism.

The abovementioned seven anthropometric traits were selected because they were the only ones for which the associated SNPs showed genome-wide significance (*P* < 5 × 10^−8^). Only data from SNPs showing *r*^2^ < 0.001 for a window size = 10,000 kb were extracted in order to ensure absence of linkage disequilibrium, as shown in Supplementary Figure 4. It was worth to emphasize that when performing MR analysis using genetic variants as instrumental variables, MR analysis needs to based on three principle assumptions (21): (1) genetic variants should be associated with the exposure; (2) genetic variants should be associated with the outcome exclusively through the exposure; and (3) genetic variants should be independent of any measured and unmeasured confounders.

The present study was considered exempt from ethics approval because it relied entirely on data from public databases that had been collected after the responsible institutions had received ethics approval and participants had given consent.

### Two-sample MR and sensitivity analyses

Effect alleles were harmonized across the genome-wide association studies, and primary MR analysis was conducted using inverse variance-weighted regression. A fixed-effect regression model was used unless heterogeneity was significant (*P* < 0.05) based on Cochran's Q statistic from inverse variance-weighted and MR-Egger regression analyses, in which case a random-effects regression model was used.

Since inverse variance-weighted regression is sensitive to invalid instrumental variables and pleiotropy (25), secondary MR analyses were performed based on MR-Egger analysis, which can detect, and correct for, possible pleiotropy (26); weighted median analysis (27); as well as maximum likelihood and penalized weighted median methods (28). The weighted median method can produce consistent causal estimates, assuming that more than half of instrumental variables reflect valid SNPs (27). Maximum likelihood and penalized weighted median methods were used to assess the robustness of MR results (28). MR analyses were performed using “TwoSampleMR” (version 0.5.6; https://mrcieu.github.io/TwoSampleMR/) in R software (version 4.0.5; www.r-project.org).

Heterogeneity was assessed in terms of Cochran's Q statistic from inverse variance-weighted and MR-Egger regression analyses. Potential horizontal pleiotropy in regression results was assessed using MR-Egger and “MR-PRESSO” (version 1.0, https://github.com/rondolab/MR-PRESSO) analyses (29) in R, with the distribution number set to 1,000. MR-PRESSO has three components: (1) detection of horizontal pleiotropy; (2) correction for horizontal pleiotropy through removal of outliers; and (3) testing of significant differences in causal estimates before and after outlier removal. Outlier SNPs that might be confounding results were identified through analysis of individual SNPs and “leave-one-out” analyses. Variance (*R*^2^) in MR refers to the proportion of total variation in the exposure that is explained by the genetic instruments. *R*^2^ for each trait was derived from the original genome-wide association studies. We verified that the *F*-statistic, defined as mean β^2^/σ^2^ across all SNPs, was at least 10 for all the anthropometric traits in our analysis in order to minimize bias from weak genetic instruments (30) (Supplementary Table 9).

## Results

**Figure 1:**
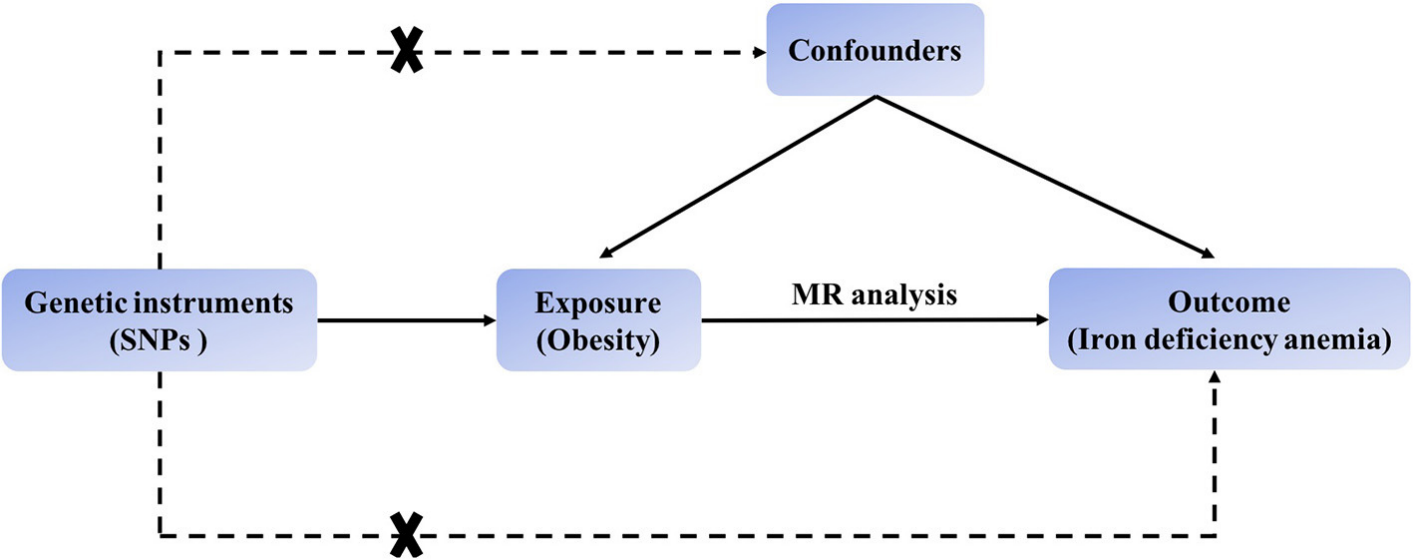
Schematic showing how Mendelian randomization was used to evaluate a causal association between obesity-related traits and iron deficiency anemia in this study.

The MR process in the present study is summarized in Figure 1. The genetic instruments initially identified to explore causal relationships between obesity-associated anthropometric traits and iron deficiency anemia are summarized in Supplementary Tables 1, 2, and the results after harmonization are shown in Supplementary Table 3. After removal of SNPs for which the allele or DNA strand was ambiguous (Supplementary Table 4), we searched for causal effects of individual obesity-associated anthropometric traits on iron deficiency anemia. The results of sensitivity analysis are shown in Supplementary Tables 6–8, and there was no horizontal pleiotropy in any of these results.

**Figure 2:**
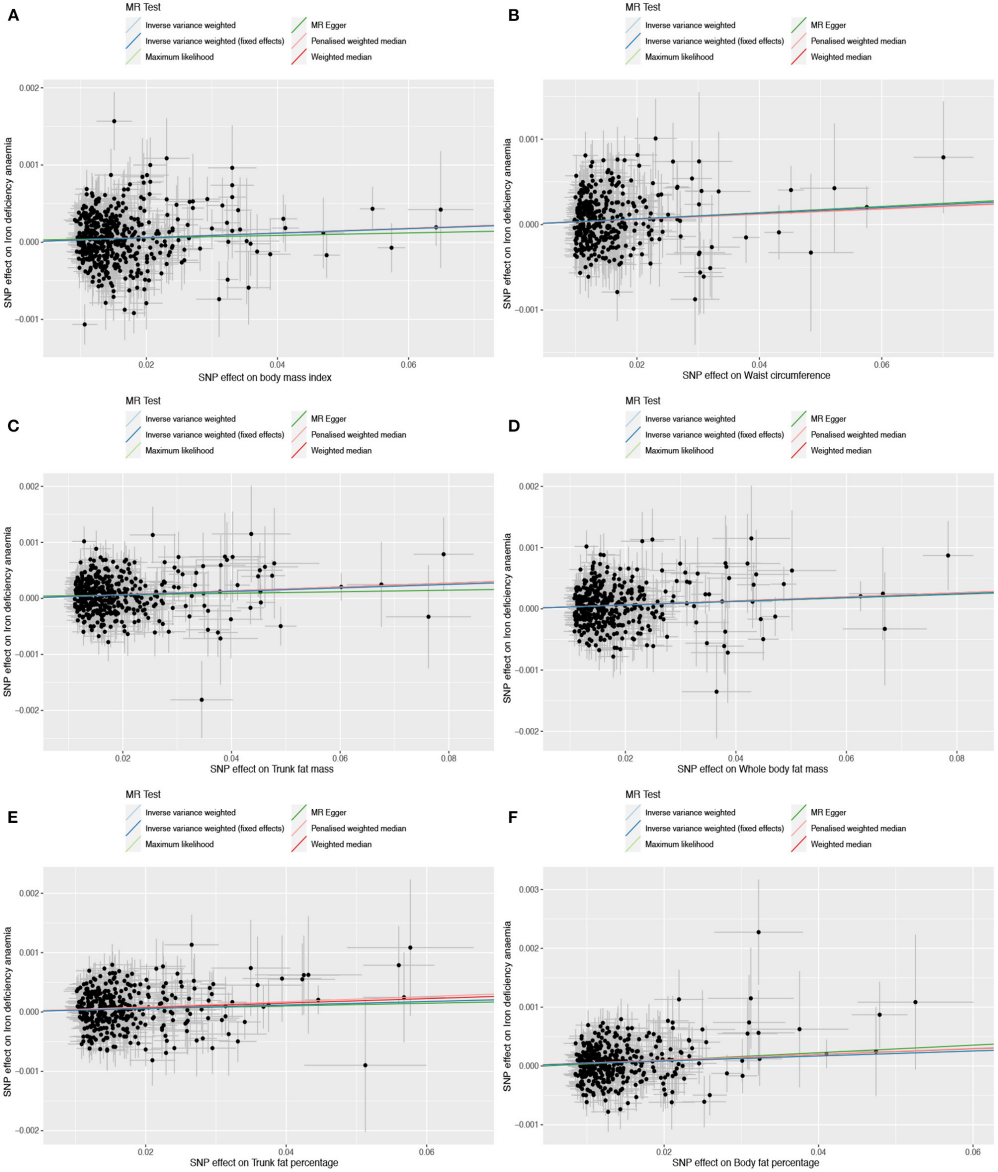
Scatter plots to assess causal associations between iron deficiency anemia and each of the following six obesity-related anthropometric traits: **(A)** body mass index, **(B)** waist circumference, **(C)** trunk fat mass, **(D)** whole-body fat mass, **(E)** trunk fat percentage, and **(F)** body fat percentage.

**Figure 3:**
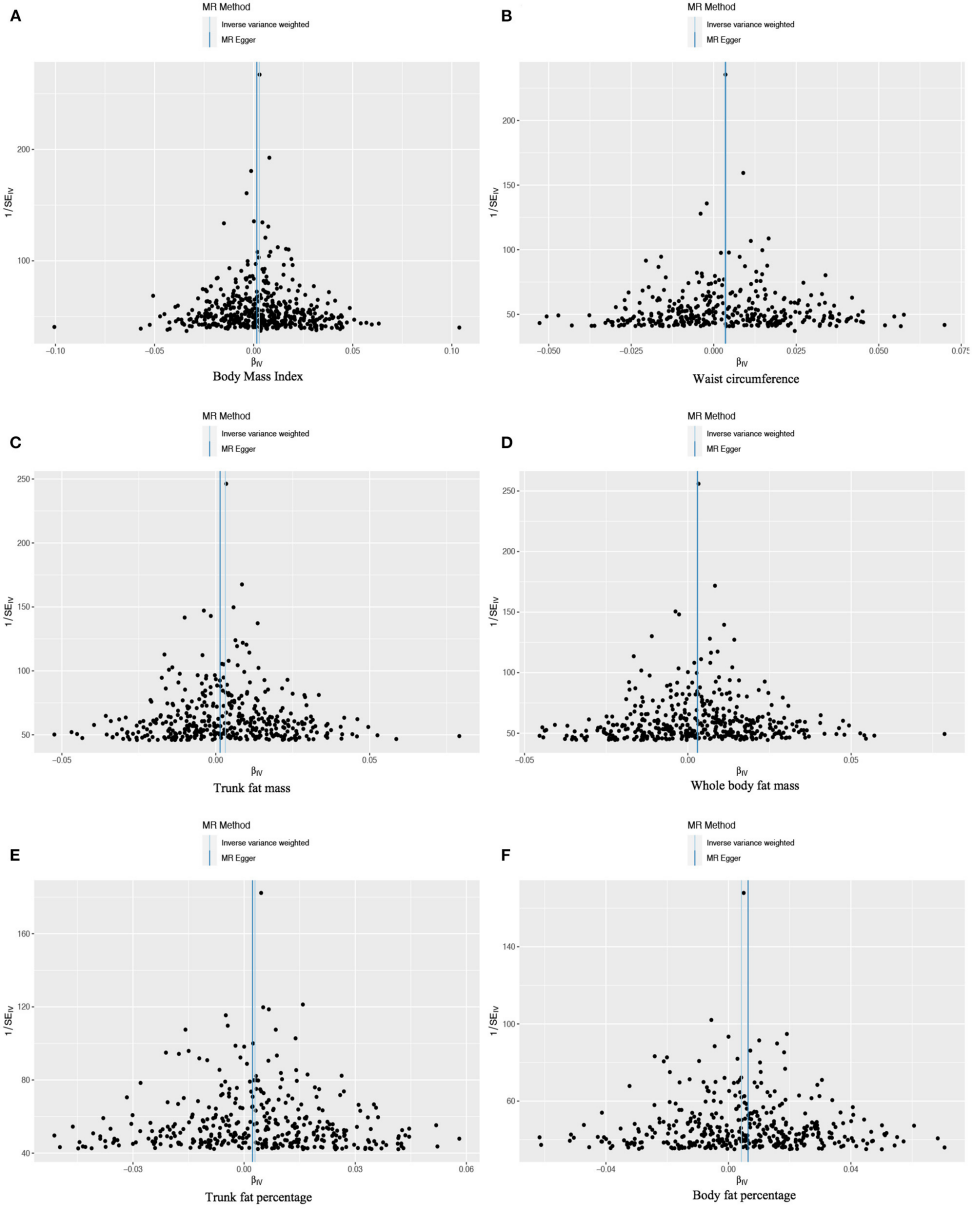
Funnel plots to assess the pleiotropy of observed causal associations between iron deficiency anemia and each of the following six obesity-related anthropometric traits: **(A)** body mass index, **(B)** waist circumference, **(C)** trunk fat mass, **(D)** whole-body fat mass, **(E)** trunk fat percentage, and **(F)** body fat percentage.

Based on 492 SNPs related to body mass index, we found a causal effect of this index on risk of iron deficiency anemia in primary and secondary MR analyses (IVW: OR = 1.003, 95% CI: 1.001–1.004, *P* < 0.001; MR-Egger: OR = 1.002, 95% CI: 0.997–1.006, *P* = 0.447; weighted median: OR = 1.003, 95% CI: 1.000–1.006, *P* = 0.029; maximum likelihood: OR = 1.003, 95% CI: 1.001–1.004, *P* < 0.001; penalized weighted median: OR = 1.003, 95% CI: 1.000–1.006, *P* = 0.023; Table 2; Supplementary Table 5). The *F*-statistic for SNPs related to body mass index was approximately 29.742 (Supplementary Table 9). The *P* values of Q statistics for inverse variance-weighted and MR-Egger analyses suggested the existence of heterogeneity (MR-Egger: Q statistic = 564.382, *P* = 0.011; IVW: Q statistic = 564.867, *P* = 0.012), so random-effects regression model was used (Supplementary Table 6). The MR-Egger intercept test suggested horizontal pleiotropy (intercept = 2.249 × 10^−5^, *P* = 0.517, Supplementary Table 7), and removal of three outliers (rs380857, rs7903146 and rs818524) in MR-PRESSO analysis did not substantially alter the original results (Supplementary Table 8). The scatter plot showed a significant positive correlation between body mass index and iron deficiency anemia, and MR intercepts were close to zero, indicating minimal horizontal pleiotropy (Figure 2A). The causal association was robust to leave-one-out sensitivity analysis (Supplementary Figure 2A) and the funnel plot was symmetrical (Figure 3A), indicating no pleiotropy. Forest plots showed the causal effect estimates between body mass index and iron deficiency anemia, and the combination of the effect estimates based on inverse variance-weighted and MR-Egger regression (Supplementary Figure 3A).

Based on 360 SNPs related to waist circumference, we found a causal effect of this parameter on risk of iron deficiency anemia in primary MR analysis with a fixed-effect regression model and in secondary MR analysis (IVW: OR = 1.003, 95% CI: 1.002–1.005, *P* < 0.001; MR-Egger: OR = 1.004, 95% CI: 0.998–1.009, *P* = 0.179; weighted median: OR = 1.003, 95% CI: 1.000–1.006, *P* = 0.063; maximum likelihood: OR = 1.003, 95% CI: 1.002–1.005, *P* < 0.001; penalized weighted median: OR = 1.003, 95% CI; 1.000–1.006, *P* = 0.077; Table 2; Supplementary Table 5). SNPs identified in iron deficiency anemia and waist circumference were found to be a available instruments, with *F*-statistics = 24.765 (Supplementary Table 9). We found no evidence of heterogeneity or pleiotropy based on MR-Egger analysis (Supplementary Tables 6, 7) and no evidence of outlier SNPs in MR-PRESSO analysis (Supplementary Table 8). The results were robust to leave-one-out analysis (Supplementary Figure 2B), and scatter, funnel and forest plots were similar to those for body mass index (Figures 2B, 3B; Supplementary Figure 3B).

Based on 404 SNPs for trunk fat mass, a causal effect of this parameter on iron deficiency anemia was found in inverse variance-weighted regression (IVW: OR = 1.003, 95% CI: 1.002–1.005, *P* < 0.001; MR-Egger: OR = 1.001, 95% CI: 0.997–1.006, *P* = 0.525; weighted median: OR = 1.003, 95% CI: 1.001–1.006, *P* = 0.007; maximum likelihood: OR = 1.003, 95% CI: 1.002–1.005, *P* < 0.001; penalized weighted median: OR = 1.003, 95% CI; 1.001–1.006, *P* = 0.008; *F* = 30.390; Table 2; Supplementary Tables 5, 9), which was conducted using a random-effects model because MR-Egger and inverse variance-weighted analyses indicated heterogeneity (MR-Egger: Q statistics = 451.735, *P* = 0.044; IVW: Q statistics = 452.489, *P* = 0.045; Supplementary Tables 5, 6). In addition, MR-PRESSO analysis did not detect significant outliers (Supplementary Table 8).

Based on 419 SNPs for whole-body fat mass, which explained 2% of its variance (Supplementary Table 9), a causal effect of this parameter on iron deficiency anemia was found in regression (IVW: OR = 1.003, 95% CI: 1.001–1.005, *P* < 0.001; *F* = 31.144), which was conducted using a random-effects model because the *P*-value of the Q statistic was < 0.05 in MR-Egger and inverse variance-weighted analyses (MR-Egger: Q statistics = 469.992, *P* = 0.037; IVW: Q statistics = 469.997, *P* = 0.040; Table 2; Supplementary Table 5). MR-Egger regression did not detect horizontal pleiotropy (intercept = 2.564 × 10^−6^, *P* = 0.949), and MR-PRESSO did not detect outliers (Supplementary Tables 6–8).

Based on 374 SNPs for trunk fat percentage, a causal effect of this parameter on iron deficiency anemia was found (IVW: OR = 1.003, 95% CI: 1.001–1.005, *P* = 0.001; *F* = 25.704; Table 2; Supplementary Tables 5, 9), and there was no heterogeneity in MR-Egger and inverse variance-weighted analyses (MR-Egger: Q statistics = 406.942, *P* = 0.103; IVW: Q statistics = 407.007, *P* = 0.109). MR-Egger regression did not detect horizontal pleiotropy (intercept: = 1.170 × 10^−5^, *P* = 0.807), and MR-PRESSO did not detect outliers (Supplementary Tables 6–8).

Based on 382 SNPs for body fat percentage, a causal effect of this parameter on iron deficiency anemia was found (IVW: OR = 1.004, 95% CI: 1.002-1.006, *P* < 0.001; *F* = 26.946; Table 2; Supplementary Tables 5, 9). No evidence of heterogeneity, horizontal pleiotropy or outliers was found (Supplementary Tables 6–8). The scatter, funnel and forest plots for trunk fat mass, whole-body fat mass, trunk fat percentage and body fat percentage were also similar to those for body mass index (Figures 2C–F, 3C–F; Supplementary Figures 3C–F).

In contrast to the other six anthropometric traits, no causal effect of hip circumference on iron deficiency anemia was detected based on 405 SNPs (IVW: OR = 1.001, 95% CI: 1.000–1.003, *P* = 0.088; *F* = 24.765; Table 2; Supplementary Tables 5, 9). These results did not reflect heterogeneity, horizontal pleiotropy or outliers (intercept = 3.480 × 10^−5^, *P* = 0.402; Supplementary Tables 6–8).

## Discussion

Using a genetic approach, this study provides evidence that obesity can cause iron deficiency anemia, based on causal relationships between this type of anemia and six well-established anthropometric traits related to obesity: body mass index (IVW: OR = 1.003, 95% CI: 1.001–1.004, *P* < 0.001), waist circumference (IVW: OR = 1.003, 95% CI: 1.002–1.005, *P* < 0.001), trunk fat mass (IVW: OR = 1.003, 95% CI: 1.002–1.005, *P* < 0.001), body fat mass (IVW: OR = 1.003, 95% CI: 1.001–1.005, *P* < 0.001), trunk fat percentage (IVW: OR = 1.003, 95% CI: 1.001–1.005, *P* = 0.001) and body fat percentage (IVW: OR = 1.004, 95% CI: 1.002–1.006, *P* < 0.001). These results were obtained in the absence of horizontal pleiotropy and outliers.

Our findings are consistent with numerous observational studies in various countries that reported associations between obesity and iron deficiency anemia (7–13). However, some studies have reported no significant association (14, 15), while at least one study has linked obesity with *lower* risk of iron deficiency anemia (17). We suggest that our finding of a causal link may be more reliable than findings from observational studies because MR analysis is less vulnerable to confounding or reverse causation. In addition, we were careful to correct for horizontal pleiotropy, in which the SNPs in the analysis might affect risk of iron deficiency anemia *via* pathways unrelated to obesity (31, 32). The consistency of our estimates from different analytical methods provides strong support for obesity as a cause of iron deficiency anemia.

Our study justifies future research to clarify how obesity may trigger this condition (17). One possibility is that the greater blood volume in obese individuals increases their iron requirement (33), which cannot be met because the diet is inadequate (7), less iron is bound to myoglobin in muscles due to low physical activity (34), genetic risk factors exist (12), and hypermetabolic medications as well as chronic inflammation reduce the level of iron available to the blood. Obesity does not appear to cause iron deficiency anemia by impairing gastrointestinal absorption (13). In fact, obese mice in one study absorbed and retained approximately twice as much iron as lean mice (33), which may indicate an adaptive response to the increase in blood volume.

Adipose tissue releases various proinflammatory cytokines called adipokines, which have been linked to obesity-related comorbidities (35). Obesity is characterized by mild chronic inflammation, which leads to the release of proinflammatory cytokines such as interleukin-6 and tumor necrosis factor-α. About a third of the circulating IL-6 is released from fat tissue (36), which stimulates the production of hepcidin through STAT3 (37). The adipokine hepcidin reduces iron output from macrophages, hepatocytes, and intestinal cells (38, 39), leading to iron sequestration within macrophages in the spleen and liver and reducing iron uptake by other cells and tissues. High levels of hepcidin have been found in obese individuals who are deficient in iron, which suggests that the iron deficiency observed in obese people may be related to mechanisms associated with hepcidin (40, 41).

## Limitations

Our findings should be interpreted with caution in light of several limitations. First, nearly all our data came from European populations, reflecting the Caucasian bias in genomic research on obesity and iron deficiency anemia. Our results should be verified and extended in other populations. Second, we cannot exclude that our analyses were confounded by intermediation effects. Third, we did not perform subgroup analyses according to sex or geographic area. These and other potential effect modifiers should be considered in future work.

## Conclusions

This two-sample MR study provides evidence of a potential causal association between obesity and iron deficiency anemia, which was robust to different analyses and rigorous pleiotropy testing. Among these anthropometric traits of obesity, people with a high body fat percentage may have a greater probability of developing iron deficiency anemia. This study can guide people to carry out scientific health management by reducing body fat percentage. Since dieting without exercise only loses muscle, not fat, people should lose weight through diet and exercise. In addition, increasing iron-rich foods is beneficial for people with iron deficiency anemia, regardless of weight. Further research should identify the molecules and pathways through which obesity can trigger iron deficiency anemia.

## Data availability statement

The original contributions presented in the study are included in the article/Supplementary material, further inquiries can be directed to the corresponding author.

## Ethics statement

Ethical review and approval was not required for the study on human participants in accordance with the local legislation and institutional requirements. Written informed consent for participation was not required for this study in accordance with the national legislation and the institutional requirements.

## Author contributions

TW and GL designed this study and drafted the manuscript. ML, JX, XL, DS, ZC, QG, YW, CW, GX, TL, RW, and JZ collected and analyzed the data. YY and MY revised the manuscript. All authors have read and approved the final manuscript.
