# Supplementary material for: Causal relationship between obesity and iron deficiency anemia: a two-sample Mendelian randomization study
Source: Front Public Health. 2023 Jun 16;11:1188246. doi: 10.3389/fpubh.2023.1188246 (PMC10313085; doi:10.3389/fpubh.2023.1188246)
Supplement: Supplementary file 1 [file Data_Sheet_1.docx]

Supplementary Materials

***Supplementary tables legends:***

**Supplemental Table 1.** Instrumental variants of considered exposures.

**Supplemental Table 2.** Extracting SNPs in the GWAS summary statistics of iron deficiency anemia.

**Supplemental Table 3.** Harmonization the results.

**Supplemental Table 4.** Identifying the palindromic and incompatible alleles.

**Supplemental Table 5.** MR analysis for the causal effects of the obesity on iron deficiency anaemia.

**Supplemental Table 6.** Heterogeneity analysis.

**Supplemental Table 7.** Horizontal pleiotropy analysis.

**Supplemental Table 8.** MR-PRESSO analysis.

**Supplemental Table 9.** *F*-statistics analysis.

***Supplementary figures legend:***

**Supplemental Figure 1.** MR analysis to assess causal associations between iron deficiency anemia and hip circumference. **(A)** scatter plot, **(B)** “leave-one-out” sensitivity analysis, **(C)** funnel plot, **(D)** forest plot.

**Supplemental Figure 2.** “Leave-one-out” sensitivity analysis to assess the robustness of observed causal associations between iron deficiency anemia and each of the following six obesity-related anthropometric traits: **(A)** body mass index, **(B)** waist circumference, **(C)** trunk fat mass, **(D)** whole-body fat mass, **(E)** trunk fat percentage, and **(F)** body fat percentage.

**Supplemental Figure 3.** Forest plots to assess the correlation between iron deficiency anemia and each of the following six obesity-related anthropometric traits: **(A)** body mass index, **(B)** waist circumference, **(C)** trunk fat mass, **(D)** whole-body fat mass, **(E)** trunk fat percentage, and **(F)** body fat percentage.

**Supplemental Figure 4.** The design flow chart for the MR analysis.
