# Supplementary figures and images for: Causal relationship between obesity and iron deficiency anemia: a two-sample Mendelian randomization study
Source: Front Public Health. 2023 Jun 16;11:1188246. doi: 10.3389/fpubh.2023.1188246 (PMC10313085; doi:10.3389/fpubh.2023.1188246)

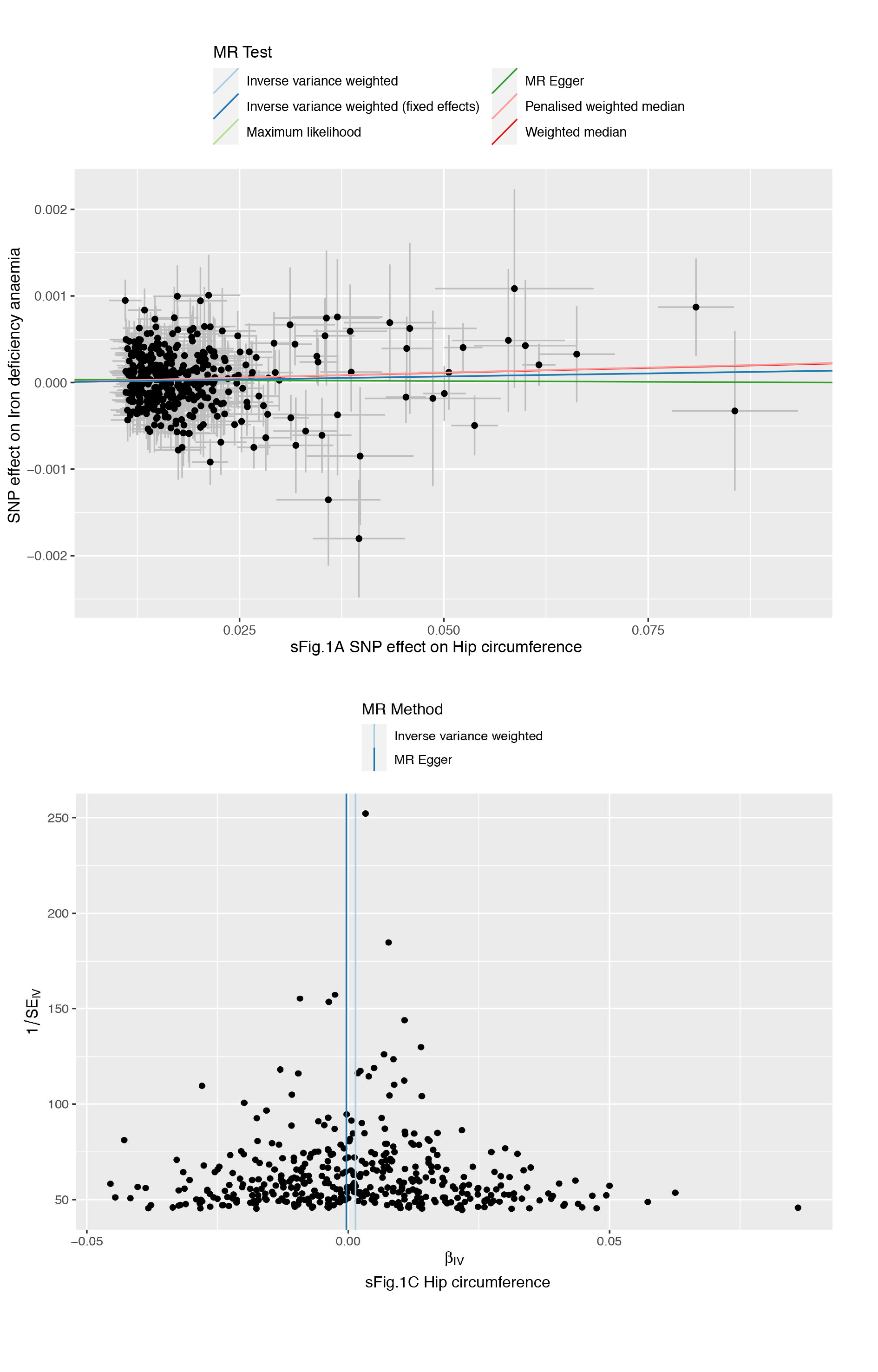

Supplement: Supplementary file 3 [file Image_1.TIF]

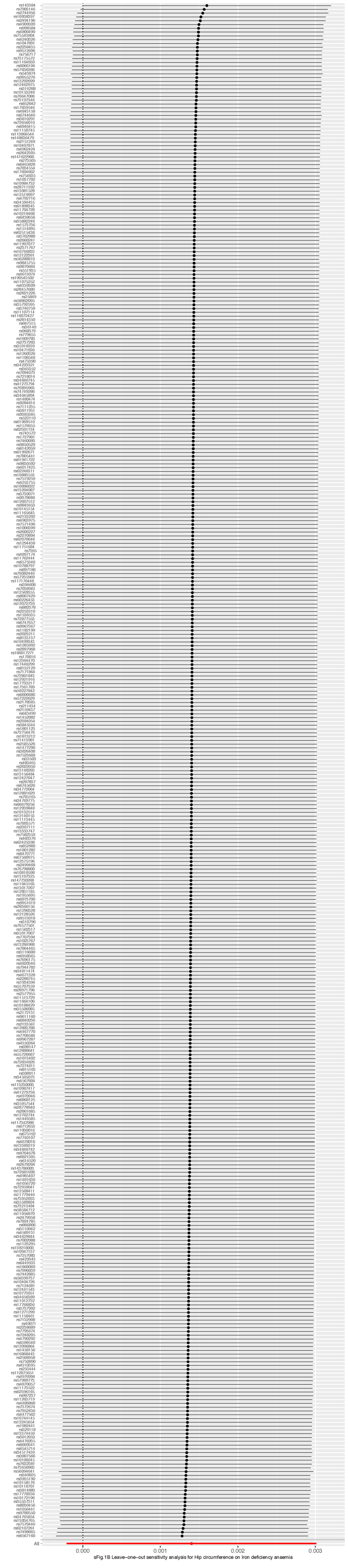

Supplement: Supplementary file 4 [file Image_2.TIFF]

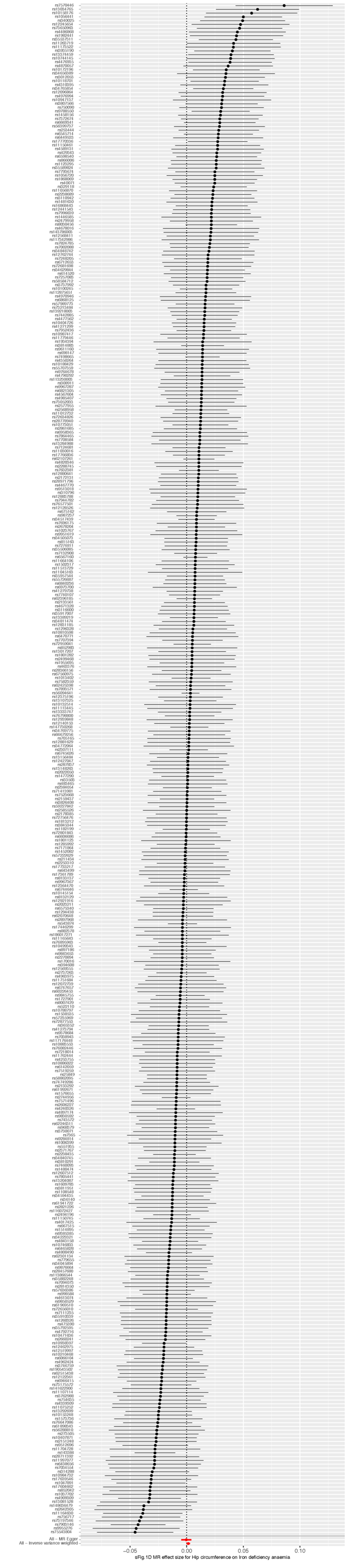

Supplement: Supplementary file 5 [file Image_3.TIFF]

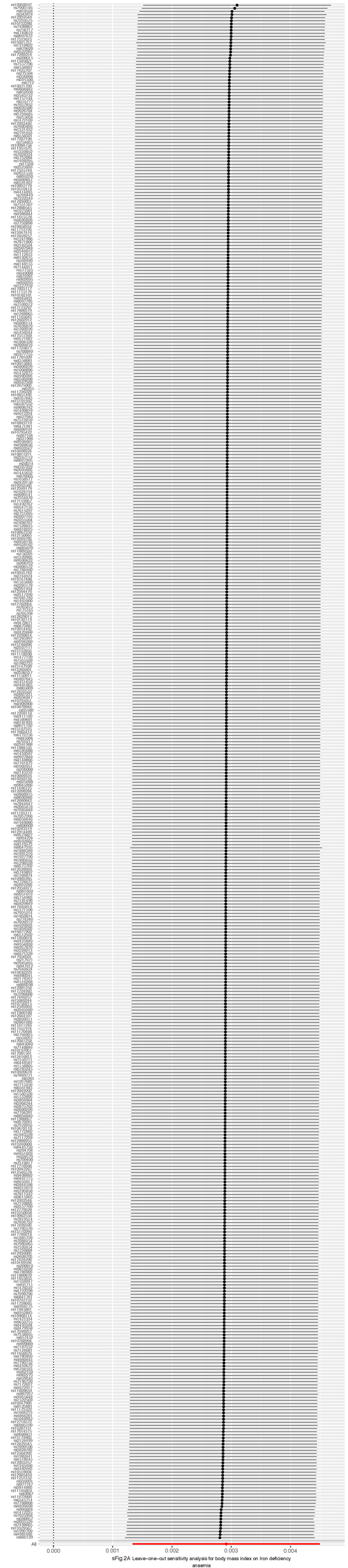

Supplement: Supplementary file 6 [file Image_4.TIFF]

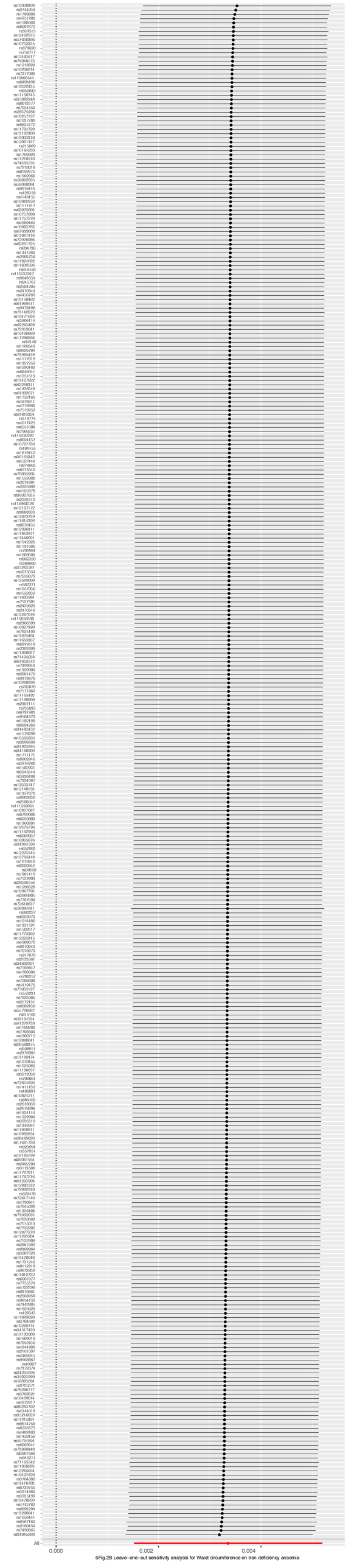

Supplement: Supplementary file 7 [file Image_5.TIFF]

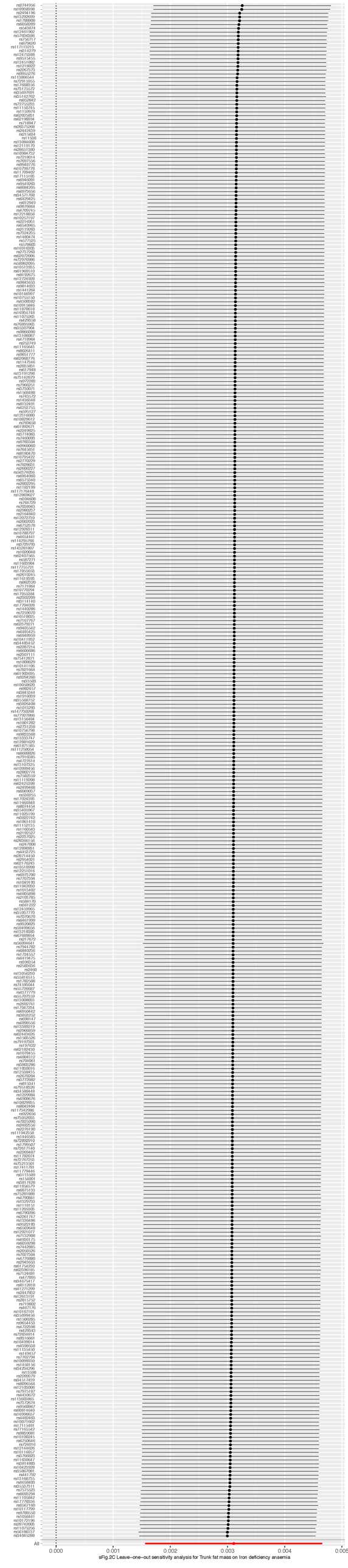

Supplement: Supplementary file 8 [file Image_6.TIFF]

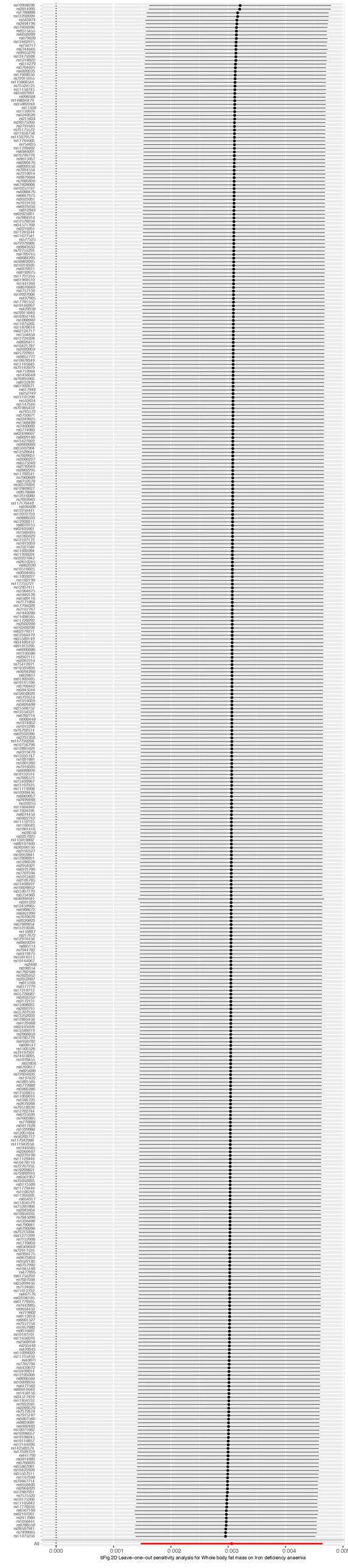

Supplement: Supplementary file 9 [file Image_7.TIFF]

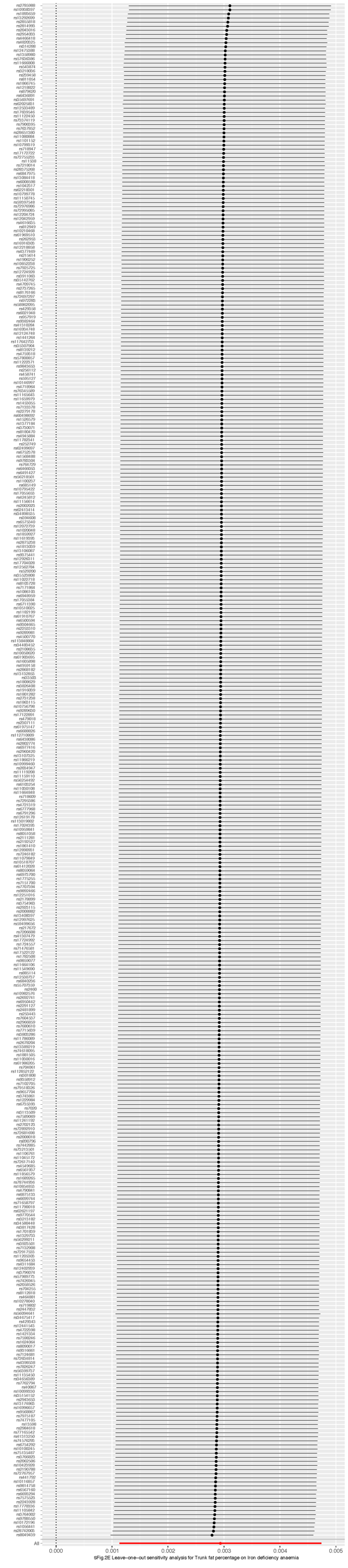

Supplement: Supplementary file 10 [file Image_8.TIFF]

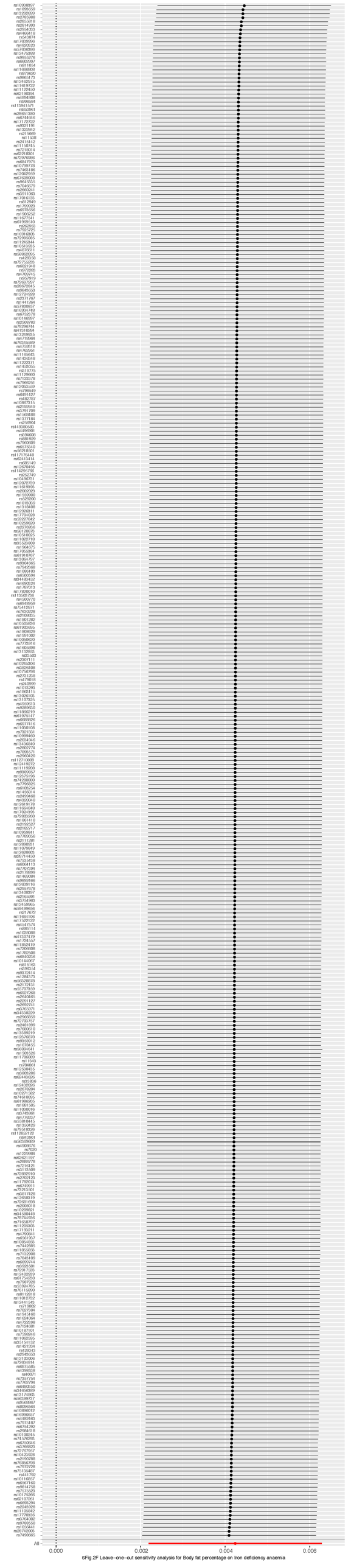

Supplement: Supplementary file 11 [file Image_9.TIFF]

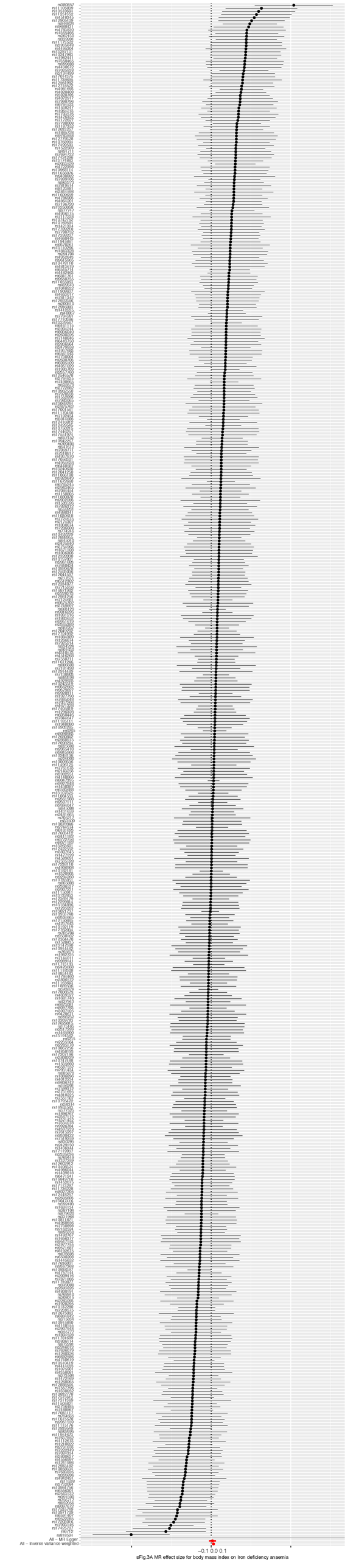

Supplement: Supplementary file 12 [file Image_10.TIFF]

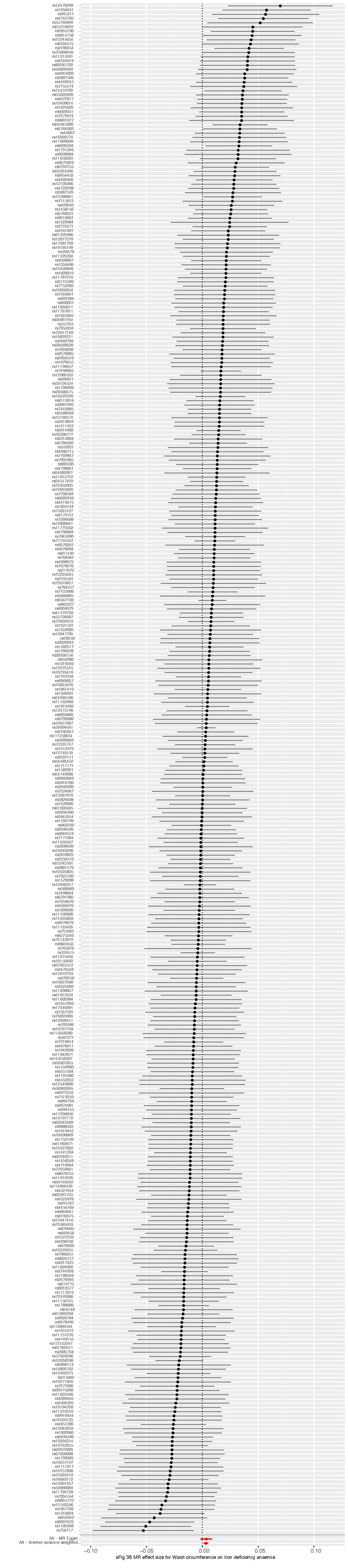

Supplement: Supplementary file 13 [file Image_11.TIFF]

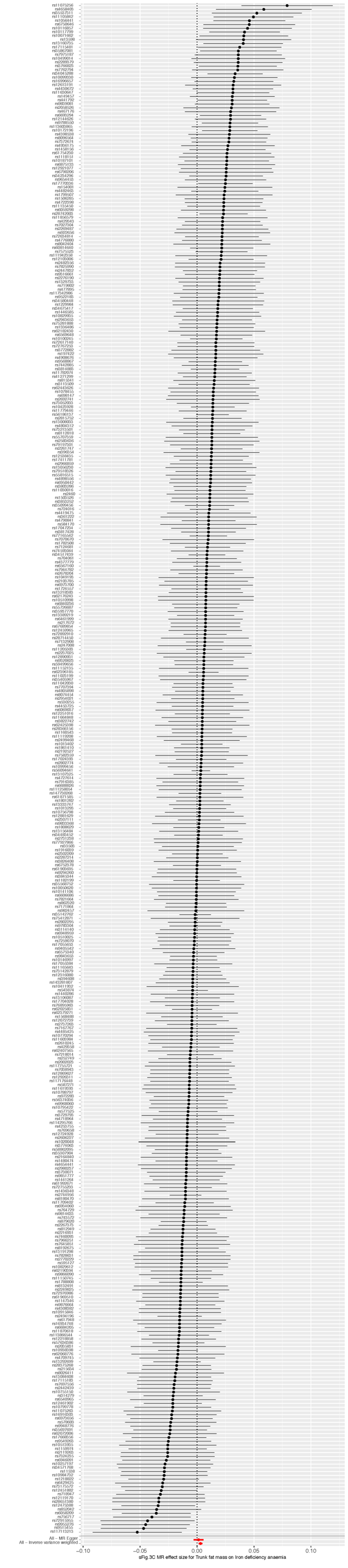

Supplement: Supplementary file 14 [file Image_12.TIFF]

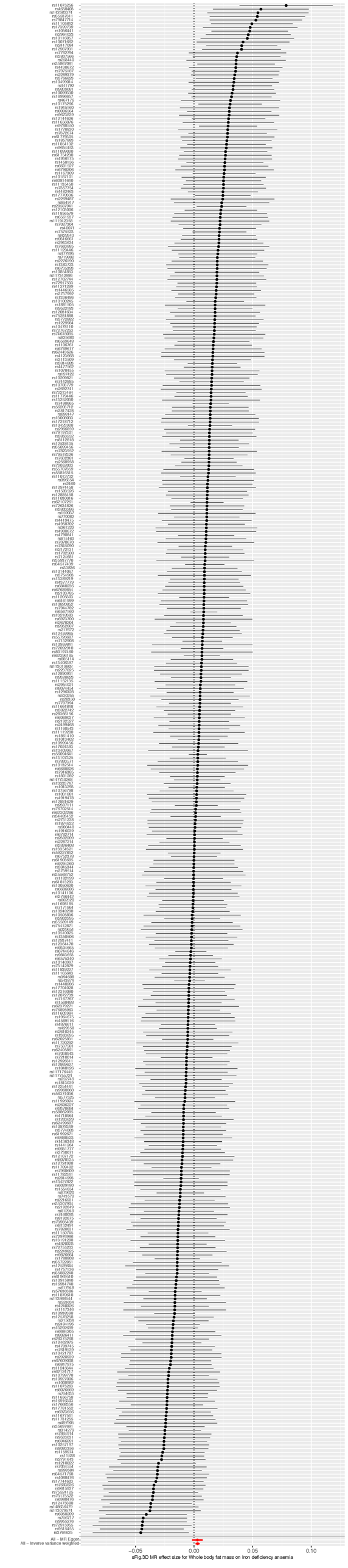

Supplement: Supplementary file 15 [file Image_13.TIFF]

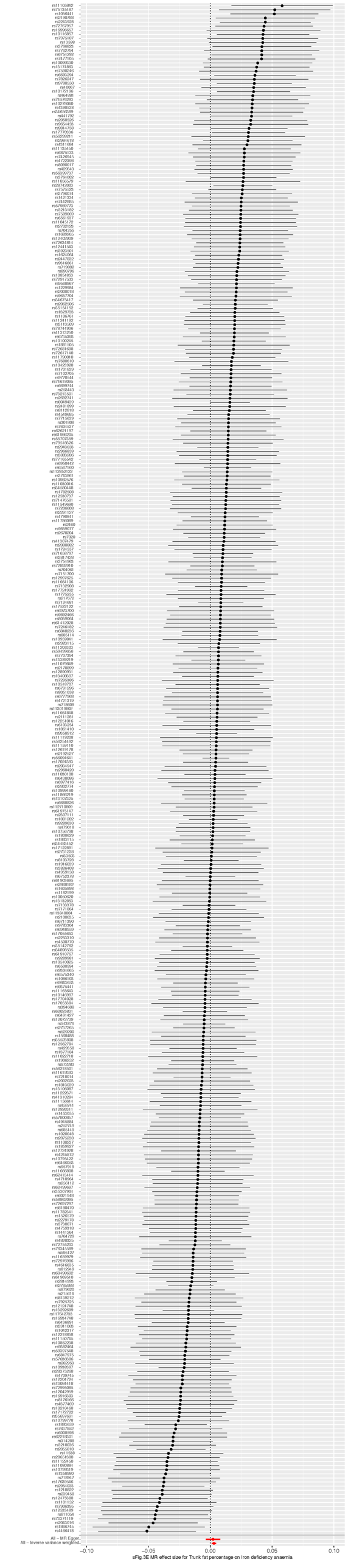

Supplement: Supplementary file 16 [file Image_14.TIFF]

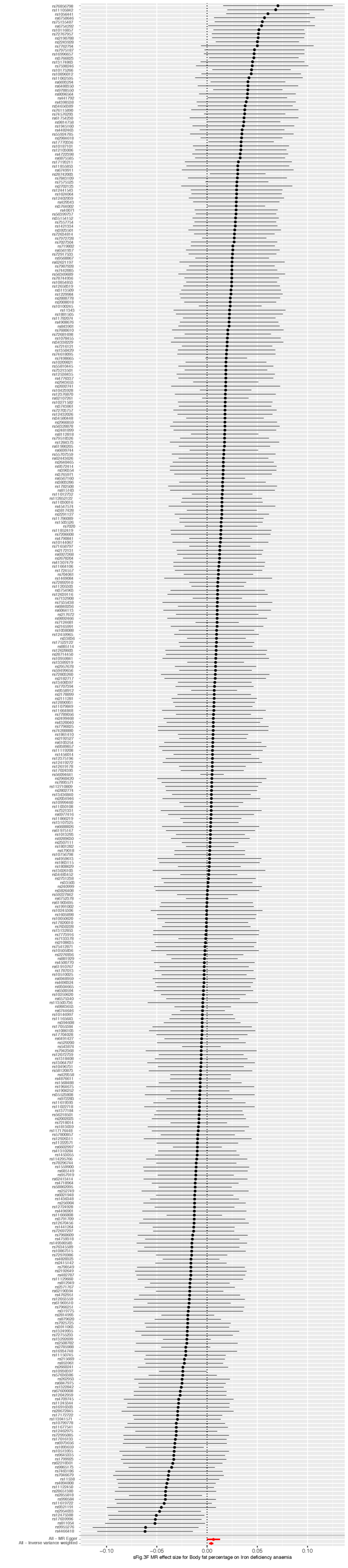

Supplement: Supplementary file 17 [file Image_15.TIFF]
